# Using Role Substitution to Improve Oral Health in Care Homes: A Process Evaluation

**DOI:** 10.1111/ger.70012

**Published:** 2025-08-07

**Authors:** Annie Hendry, Sarah R. Baker, Gerry McKenna, Georgios Tsakos, Ivor Chestnutt, Craig Smith, Ciaran O'Neill, Alison Jenkins, Rachel Evans, Saif Sayeed Syed, Michelle Harvey, Anup Karki, Kirstie Moons, Fiona Sandom, Michael Donaldson, Caroline Lappin, Karen Shepherd, Lorraine Morgan, Paul R. Brocklehurst

**Affiliations:** ^1^ Bangor Institute of Health and Medical Research Bangor University Bangor UK; ^2^ Department of Oral Health, Dentistry and Society University of Sheffield Bangor UK; ^3^ Centre for Public Health Queens University Belfast Belfast UK; ^4^ Department of Epidemiology and Public Health University College London London UK; ^5^ College of Biomedical and Life Sciences Cardiff University Cardiff UK; ^6^ Division of Cardiovascular Sciences University of Manchester Manchester UK; ^7^ NWORTH Clinical Trials Unit Bangor University Belfast UK; ^8^ Primary Care Division Public Health UK Leicester UK; ^9^ Health Education and Improvement Wales UK; ^10^ Dental Services Northern Ireland Health and Social Care Board Belfast UK; ^11^ Community Dental Services South Eastern Trust Northern Ireland UK; ^12^ Patient and Public Involvement Oxford UK

**Keywords:** care home residents, complex interventions, implementation, oral health, process evaluation, randomised controlled trials

## Abstract

**Background:**

The oral health of many older adults residing in care homes is poor and service provision is limited. Role substitution has been suggested as a potential model to improve service provision in this context and describes the reallocation of tasks from a dentist to other members of the dental team.

**Objectives:**

To undertake a theoretically informed process evaluation alongside a pragmatic cluster‐randomised controlled trial to determine whether the use of Dental Therapists and Dental Nurses could improve the oral health of dependent older adults in care homes in the UK.

**Materials and Methods:**

Semistructured interviews were held with 17 key stakeholders responsible for intervention delivery. Parallel observations were utilised during the intervention delivery phase in 22 homes. Both were conducted inductively using the main themes from the Promoting Action on Research Implementation in Health Services (PARIHS) framework to focus on intervention delivery and implementation.

**Results:**

Stakeholders were receptive to the potential of using role substitution in this setting and saw this as a viable alternative to current practice. Partnership working was considered key, but was not always observed, and some care staff did not see oral health as their responsibility. The physical environment of the care home setting created a number of challenges, and sugary food and drinks were ubiquitous and formed an important part of the day‐to‐day structure within the home.

**Conclusion:**

Although role substitution has the potential to meet the needs of dependent older people, a number of challenges exist in promoting oral health and delivering service provision.

## 1 Background

In the United Kingdom, a Dental Therapist is a registered dental professional who is able to undertake a clinical examination, provide supragingival and subgingival debridement, and carry out direct restorations under analgesia. A Dental Nurse (DN) is also a registered dental professional who is able to provide oral health education, oral health promotion, and apply topical fluoride (following appropriate training). Evidence from the United Kingdom (UK) suggests that DTs and DNs could help address future oral health needs [1, 2] including the care for dependent older people residing in care homes [3]. This is important given the high level of dental need experienced by the latter [4, 5] and the poor level of service provision in the UK [6]. Evidence from recent reviews suggests that role substitution is likely to be a key factor in meeting the growing demand for health care and has the potential to increase access to care, care quality, and continuity of service provision [7, 8]. Although a large proportion of need in care homes could be wholly provided by DTs, the underlying empirical evidence is limited [3, 9]. In a recent systematic review of oral health interventions for older people in care homes, many of the 30 included studies were judged to be of poor quality, lacked precision, and were of limited duration and follow‐up [10]. No included studies featured role substitution as the primary model of care.

‘uSing rolE substitutioN In care homes to improve oRal health’ (SENIOR) was a pragmatic cluster‐randomised controlled trial to determine whether DTs and DNs could improve the oral health of dentate older adults over 65 years‐of‐age residing in care homes [9]. Over a 6‐month period 43 care homes and 284 residents were recruited across London, Northern Ireland, Northwest of England and Wales. DTs in the active arm (*n* = 22 care homes) provided routine dental care and DNs professionally administered fluoride varnish, oversaw the use of high‐strength fluoride toothpaste (5000 ppm), supervised toothbrushing and acted to facilitate the delivery of oral health messages in the setting, using materials developed from an accompanying study based on NICE guidelines [11]. This was compared to ‘treatment as usual’ in the control arm (*n* = 21 care homes).

The aim of this study was to utilise the ‘Promoting Action on Research Implementation in Health Services’ (PARIHS) framework [12] to conduct a process evaluation of the SENIOR trial, focusing on the delivery of the intervention and the factors that could facilitate its adoption in practice. This built on the findings of an earlier paper that focused on the care home context [13]. The interaction between these contextual issues (e.g., care home pressures, values and beliefs of staff towards oral health), factors related to the individual resident (e.g., ability to self‐care, level of cognitive awareness) and clinical challenges in providing care, adds substantive complexity to the delivery of any intervention in this setting [6, 9]. This makes the study of how these different factors manifest themselves important, including how they are enacted within the physical space of a care home. These factors have been articulated recently in another care home study that used a ‘system‐lens’ approach [14].

## 2 Methods

### 2.1 Ethical Considerations

This study was reviewed and granted full ethical approval by the Bangor University School of Health Sciences Ethics Committee (2021–16,773).

### 2.2 Sampling and Data Collection

Stakeholders directly involved in the delivery of the intervention were invited to participate. The range and number of stakeholders that were interviewed are provided in Table 1, but included DTs, DNs and care home managers and staff. The sampling and data collection process was similar to the approach described in Hendry et al. [13]. Audio‐taped interviews were conducted and recorded with virtual platforms (*n* = 17) and observational work was face to face (31 residents receiving the intervention across 7 care homes). In the latter cases, an observational template was used to augment the interview data. All participants were given a Participant Information Sheet and were asked to provide informed written consent prior to the interview. Each interview lasted between 30 and 60 min. Data were anonymised, fully transcribed and analysed thematically by the same researcher (AH).

**TABLE 1:** Participants of the process evaluations.

Participant	Role	Region
#1	Dentist	London
#2	Clinical Lead for services	NW England
#3	Clinical Lead for services	ABUHB
#4	Dental Nurse	ABUHB
#5	Dentist	ABUHB
#6	Dental Nurse	London
#7	Dental Nurse	London
#8	Dental Nurse	NW England
#9	Dental Therapist	London
#10	Dental Therapist	Northern Ireland
#11	Dental Nurse	Northern Ireland
#12	Dental Nurse	Northern Ireland
#13	Care home manager	London
#14	Dental Therapist	Northern Ireland
#15	Dental Nurse	Northern Ireland
#16	Dental Therapist	NW England
#17	Dentist	BCUHB

### 2.3 Data Analysis

Both the semistructured interviews and the observational data were coded using thematic analysis, which involved a flexible, interpretive approach to facilitate the identification of themes [15]. The transcripts were individually coded and themes were mapped across to the PARIHS framework and augmented by the observational template to understand how the intervention was employed in the care homes.

### 2.4 Theoretical Approach

PARIHS is an implementation framework that has been extensively utilised in health services research [12, 16]. It posits that successful implementation is largely a function of a receptive context, organisational culture, leadership, and facilitation [16]. The nature of the setting was explored in detail in Hendry et al. [13] whilst this study sought to understand the factors that influenced the delivery of the intervention and its adoption in practice.

To expedite this further, the face‐to‐face interview data were augmented by observational data. This enabled the research team to, in part, address the methodological challenges reported in the earlier paper, where it had been difficult to meaningfully engage with residents to understand their own perspective on oral health, given the level of cognitive impairment of many of the participating residents.

Spradley (2016) proposes that observations should focus on a number of broad dimensions, which broadly align with the key elements of the PARIHS framework [17]. These include: 'space'—the physical setting, such as rooms and location; 'actors'—the people involved in the interaction/intervention; 'activities'—the activities conducted by the actors; ‘objects'—the physical elements involved in the activities and space used by the actors; 'time'—the sequencing of activities and objects; 'goals'—what the actors seek to accomplish; and 'feelings'—what emotions the actors express [17].

To simplify the process of capturing observations within the context of a busy care home, a bespoke template was created (Table 2), which aligned Spradley's key dimensions with PARIHS. This enabled field notes to be made during the semistructured interviews and an understanding of how different factors (the physical space, actors and objects/resources) interacted with each other within the setting.

**TABLE 2:** Adapted framework to augment the semistructured interviews.

Care home:	Location in care home:	Time point:

	Context	Facilitation
Physical space (room shape/layout)	What is the space?	How is the space affecting the intervention?
Actors (residents/staff/managers)	Who are they?	What are the actors doing (activities)?
Objects/resources (intervention/biscuits)	*What objects do you see?*	*How do objects/resources affect the delivery of the intervention?*

*Note:* 1: Initiative fit, 2: Receptiveness to change, 3: Responsibility, power and authority, 4: Resource allocation, 5: Relationships, 6: Prevailing beliefs of stakeholders, 7: Staff turnover, limited time and training gaps, 8: Training and leadership, 9: Role clarity and consistency, 10: Organisational structures and access, 11: Enabling and empowering.

## 3 Results

Ten codes were generated and Table 3 highlights how these were mapped onto the main themes of PARIHS: Receptive Context; Culture; Leadership and Facilitation. The codes generated were: (1) Receptiveness to role substitution; (2) Importance of care home managers; (3) Financial resources; (4) Resource allocation for sustained implementation; (5) Co‐working between dental professionals and care home staff; (6) Relationships between DTs, DNs and residents; (7) High sugar culture in care homes; (8) Training needs of care home staff; (9) Challenges of the physical environment; (10) Liaison among Dentists, DTs and DNs; and are described in order below.

**TABLE 3:** Coding frame based on Parihs.

Parihs elements	Parihs criteria	Codes elicited*
Receptive context	Receptiveness to change	1: Receptiveness to role substitution (use of DTs and DNs)
	Decision‐making, power and authority processes	2: Importance of care home managers
	Resources allocated and feedback provided	3: Financial resources 4: Resource allocation for sustained implementation of the intervention
	Clearly acknowledged boundaries	5: Co‐working between dental professionals and care home staff
Culture	Prevailing values/beliefs	7: High sugar culture in care homes
	Consistency of individuals role/experiences	5: Co‐working between dental professionals and care home staff
	Promotes learning organisation	8: Training needs of care home staff
Leadership	Transformational leadership, role clarity and effective teamwork	5: Co‐working between dental professionals and care home staff
	Organisational structures	9: The challenge of the physical environment
	Enabling/empowering approach to decision‐making/teaching/learning	2: Importance of care home managers
Facilitation	Using roles to enable others	2: Importance of care home managers 5: Co‐working between dental professionals and care home staff 6: Relationships between the Dental Therapists and Dental Nurses and the resident 10: Liaison between Dentists, Dental Therapists and Dental Nurses
	Using skills and attributes to enable others	2: Importance of care home managers 5: Co‐working between dental professionals and care home staff 6: Relationships between the Dental Therapists and Dental Nurses and the resident 10: Liaison among Dentists, DTs and DNs

* Some codes cover multiple elements within PARIHS.

### 3.1 Receptiveness to Role Substitution (Use of DTs and DNs)

The majority of stakeholders were very positive about the use of DTs and DNs in a care home context. Care home managers argued that it offered a model to increase access to care. The DTs and DNs reported that it gave them a sense of autonomy in their clinical practice. Equally, they reported that they particularly enjoyed the experience of working in the care homes setting.

I've really enjoyed this past six months, it's given me free range to use all my knowledge that I've learnt over the time I've been a dental nurse (Dental Nurse, London)

‘It's opened a whole new world for me, because I've always been under a directive of a Dentist in clinic, but this way I'm out on my own: arranging my own time; arranging how I see the residents (Dental Nurse, London)

### 3.2 Importance of Care Home Managers

Care home managers were pivotal in facilitating intervention delivery, given their role in running the care home and setting the culture (e.g., clinical protocols, communication strategies, staffing ratios). In care homes where there was less engagement with the SENIOR trial, intervention delivery was made more difficult.

I always say a care home is only as good as the manager that manages it. (Clinical Lead for services, Wales)

There's not really anyone that's sort of taking charge of it, I'll be honest. Or it might be a care home manager but they're not actually involved in the day to day looking after the residents in terms of brushing. (Dental Therapist, London)

### 3.3 Financial Resources

Many stakeholders argued that the financial resources within care homes were an issue. Care home managers explained that many residents were funded by local authority arrangements and this meant that they could not afford the costs associated with dental care. DTs and DNs found that some residents did not have sufficient financial resources to pay for oral health care items that would normally be recommended to improve oral cleanliness, for example, toothbrushes and toothpaste. In some of the observations, DTs and DNs were seen to provide the residents with these items.

And most of my residents are local authority funded or NHS funded [where we are] is not like the suburbs where people have got loads of money. (Care Home Manager, London)

Who buys their toothbrushes? Who goes out and buys them? (Dental Nurse, London)

### 3.4 Resource Allocation for Sustained Implementation of the Intervention

Some of the DTs and DNs raised doubts regarding the availability of dental staff for long‐term delivery given the amount of time the intervention took from day‐to‐day clinical diaries. Time spent in a care home was time spent away from the clinic. Equally, providing care within this setting created additional demands and required both administrative and travel time to be factored into service delivery. Observations highlighted the difficulties with care provision, as DTs and DNs had to fit in with the rhythm of the care home such as working around meal times, residents’ appointments and activities.

I've only taken on the two homes so far, so it hasn't had a huge impact, but still does take up usually a whole day of my admin time when I go, and it means taking all the resources home with me. (Dental Nurse, London)

So we book our administrative time me for the day afterwards so I can do all of this. And like chasing referrals and trying to find out answers. So I think actually if they're trying to account for the time and resource spent, it's a lot more than what's been recorded on those forms. (Dental Therapist, London)

### 3.5 Co‐Working Between Dental Professionals and Care Home Staff

Good working relationships between dental professionals and carers were seen to be critical in facilitating intervention delivery. However, this was not observed in all the care homes and communication between care home managers and staff about the intervention was not always clear. In some care homes, care staff were not aware of the trial and would limit their involvement in the intervention to showing DTs and DNs to the residents' room. Equally, many of the care home staff did not see oral health as their responsibility.

I found that no matter what time you go, whenever you knock the door, they say “I didn't know you were coming or no‐one knew. (Dental Therapist, Northern Ireland)

It would have been nice for someone who knows the residents well to take me in and just introduce me ⋯. I felt very much abandoned by them, it was like, get on with it. (Dental Nurse, Wales)

### 3.6 Relationships Between the DT and DNs and the Resident

Good relationships between the individual resident and the dental team were also key. DTs and DNs explained that they liked to spend time getting to know the resident, finding ways to communicate with the resident to make intervention delivery easier.

However, observations highlighted how dementia and variations in cognitive awareness made clinical care more difficult and how this could vary day to day with the same resident.

We just have to try and do the best we can on the day. And sometimes that isn't anything at all, because we can't get near them (Clinical Lead for services, NW England)

And with the majority of them, it's just so challenging, even for the carers, because they just wouldn't even open their mouths. (Dental Nurse, London)

### 3.7 High Sugar Culture in Care Homes

The consumption of sugar‐rich food and drink was very much a part of the care home culture. Observations revealed that daily routines in care homes were often built around meal times and cups of tea with cake or biscuits.

I'd say the nursing home is quite structured as in that they get a breakfast, a morning scone or whatever, lunch, an afternoon snack, dinner and a supper. (Dental Nurse, Northern Ireland)

DNs reported difficulties in finding suitable ways to talk about sugar and healthy eating, given how engrained it was in everyone's day. It appeared that many residents did not have much choice in what they ate or drank. One way to reduce this level of consumption is targeted dietary advice to care home managers, but sweets and chocolates were often brought in by family members. There was also a feeling that tea and cake time was one of the few things that the residents were looking forward in a daily routine and so it was hard to tell them they shouldn't have it.

We were looking for the gentleman's toothpaste and the drawers were just full of chocolates and biscuits that the family bring in, I just thought, how are you going to win that battle there. (Dental Nurse, North West England)

Observations noted that the most ubiquitous commodity was fruit juice. Almost all residents had jugs of juice in their rooms. One resident was in the middle of their breakfast when the DT and DN arrived and said they would finish it after the dental team had left. In this case, the DT was unable to clean their teeth due to the level of oral debris. After the intervention was completed, the first thing the resident asked for was ‘a nice glass of orange’. In another observation, the DT used the presence of a jug of fruit juice as an opportunity to talk about the hidden sugar in the drink. The resident told the DT that the juice had ‘no added sugar’. In yet another observation, the carer's face dropped when she was told that resident couldn't eat for 45 min after a fluoride varnish application, given that meal times were approaching and that this would then create a problem in the management of the day. During one observation, the resident was advised not to eat or drink for half an hour following the fluoride varnish application; however, 10 min later, she was in the lounge having tea and cake. One DN told the carers that she was going to move a resident's drink away from him so he couldn't drink it following the fluoride application.

A lot of them will keep squash in, especially when it's warm, to keep the residents hydrated because they don't always want cups of tea. (Dental Therapist, North West England)

I said, try to reduce the frequency of sugar and maybe get a sugar free diet or alternative if possible. And I've got a carer saying, well, that's very expensive and we cannot afford to pay for that. (Dental Therapist, Northern Ireland)

### 3.8 Training Needs of Care Home Staff

The dental professionals highlighted the need for care home staff to be trained in improving oral health. This is important as observations revealed that some of the residents were clearly unable to brush their own teeth without any assistance. Lack of mobility compounded this issue as many residents were in their bed with the toothbrush and the sink was across the room.

But what I'm finding is that actually when the advice goes to someone that can't brush for themselves, or maybe they need help getting to the bathroom even, or they need help to brush for them, that's where it's not done and that's where I'm not seeing any improvement. (Dental Therapist, London)

The materials that were supplied as part of the intervention appeared to be received well, but only added to the burden of clinical paperwork that was observed in the care homes. Equally, some of the DTs and DNs had concerns that the oral health information was not reaching all care staff, particularly those on night shifts. In one home, the DN had displayed the checklists on each resident's bathroom door, but these were completed inconsistently by the carers. Recommended brushes were not always in the resident's bathrooms and Duraphat toothpaste was not always used.

I mean, the care plan I thought looked really good, but I think all the other associated documentation may be a bit like, woah, have I got time to do all this? (Clinical Lead for services, Wales)

It all looks very comprehensive to us and it looks easy to complete, but if it's just another set of paperwork that you've got to complete on top of other things that you're doing I can see it gets missed. (Dental Nurse, London)

### 3.9 The Challenge of the Physical Environment

Performing clinical tasks in a care home environment was highlighted as a challenge for the dental team. Observations recorded DTs and DNs trying to make the most of the physical space, often with no table space for their equipment. Lighting was another issue, and some of the DTs and DNs had to rely on torches. In some cases, the DTs and DNs worked together to make the best use of space. Some residents were in adjustable beds, which made things easier, but the use of armchairs and wheelchairs required the dental team to kneel, crouch, or bend across the resident to see into the mouth.

Participants reported that there were limitations to tasks given in the care home setting that they could only do hand scaling and basic fillings and that more complex treatment would require the patient to be seen in the clinic. The application of the fluoride varnish was described as easy to do in the care homes and the DN's were confident in doing this element.

We're really just patching people up. If we can do simple fillings that don't need any drilling, dressings basically and scaling and polishing, that kind of thing. (CDS lead, North West England)

Fluoride is very easy to put on, so… The only problem is if the mouth is full of food, if they've just eaten or plaque, that'll be the only thing. (Dental Nurse, North West England)

### 3.10 Liaison Among Dentists, DTs and DNs

A good working relationship between the dentist and the DTs and DNs was seen as key in terms of managing care that was beyond the Scope of Practice of the DT or DN and in terms of prescribing medicines. Some of the dentists interviewed suggested that many patients require management that lies beyond the Scope of Practice of DTs and DNs.

My impression is that a lot of patients need treatment that is beyond their scope, so a lot of patients need teeth taking out or dentures, which obviously a therapist can't do. (Dentist, London)

## 4 Discussion

A process evaluation was undertaken alongside an empirical trial to understand the contextual factors that influenced intervention uptake, delivery, and implementation [18, 19, 20].

The active involvement of care home managers and staff appeared critical in enabling the delivery of the intervention by DTs and DNs [14, 21]. Although many appear to be aware of the importance of oral health, they were not always aware of best practices in promoting prevention [21]. This finding concurs with a recent report by the Care Quality Commission in England, the body which oversees the regulation of care homes in England. Whilst noting an increased awareness of the importance of oral health among care home managers, there remained a varying amount of detail found in the resident's care plans [22]. Equally, some care home staff did not see oral health as part of their role, a role which was ascribed to the visiting DTs and DNs. This suggests that it is critical to remind all care staff of their role in relation to oral health and to make this a specific element of the monitoring process by the different regulators across the United Kingdom. The provision of materials to help this understanding was welcomed but only added to the amount of paperwork that was required to be completed by care home staff for each resident [14].

Facilitation and co‐working between dental and care home staff was also key to delivering a model of care based on role substitution. Goodman et al. (2017) have highlighted the importance of aligning the goals of the different staff, and Patel & Gallagher (2024) stress the importance of partnership working [21, 23]. In this study, integrated approaches were not always forthcoming, and in some cases, dental staff were left to manage the residents on their own. Delivering the intervention in the physical environment of the care home setting was also a challenge and aligns with Patel et al. (2019) and Patel & Gallagher (2024) [21, 23].

The excessive consumption of sugar within the care home and how this was integrated into care home culture poses a substantive problem [6, 14]. Both the interviews and observations highlighted how this formed part of the day‐to‐day structure and how ubiquitous its availability was. Residents are commonly encouraged to drink small quantities during the day to maintain hydration and ensure that enough calories are consumed [21]. However, this can dramatically increase caries risk status and could potentially offset any advantage provided by the use of topical fluorides, given the frequency and volume of sugar consumption.

Many residents were reported to have significant levels of plaque on arrival at the home, and those that were not already prioritising their own oral health prior to entry appeared to be more resistant to oral care. Equally, a lot of residents were no longer able to care for their own teeth and relied on personal care from staff. Observations found that staff were under considerable pressure to undertake other care duties and were often working in an environment with a high level of turnover. Similar findings have been found in other studies in the United Kingdom [6, 23, 24]. Many residents also lacked the financial resources to purchase products to improve their oral health, which was also found by Gopalakrishnan et al. (2019), leading to DTs and DNs providing toothbrushes and adding to the potential cost of service provision, should this model be adopted [25].

Despite these challenges, the potential for role substitution in this context appeared to be positive and aligns to findings from earlier studies [1, 2, 7, 8]. Both DTs and DNs enjoyed the increased autonomy to manage their own workload, work independently and use their full Scope of Practice. In the care home context, both DTs and DNs reported enjoying working with older people and drew personal value from providing care for them. Mutsekwa et al. (2024) argue that role substitution requires supportive organisational cultures, adequate resources, leadership commitment and peer endorsement [7]. Thorne et al. (2001) argue that the successful implementation of oral health programmes within a care home environment is reliant on defining: 1—a clearly articulated service plan; 2—awareness of and commitment to oral health among the staff; and 3—shared understandings between staff and the administration [26]. Whilst many of these factors were evident during the study, sustained implementation of this model of care also requires critical decisions about suitable funding models and acceptance by clinical leaders that working in care homes is not as efficient when compared to providing care in the clinic [7, 8]. Current service provision in care homes across the United Kingdom is poor, and there has been a continuing reduction in the supply of services for over a decade, with no national specifications for service provision [4, 21]. Role substitution offers much promise in this context; yet sustained implementation of this model of service provision requires substantive and ongoing financial support [7, 21].

The inclusion of a well‐conducted process evaluation alongside an empirical trial was key to understanding the factors that facilitate or impede intervention delivery [18, 19]. Recent studies in dentistry in the United Kingdom have highlighted the value of these parallel studies to understand important contextual factors and pathways to impact [14, 20]. Both the interviews and the observations highlighted the complexity of the care home context, factors related to the individual resident and many clinical challenges in providing care. This concurs with earlier studies undertaken in the United Kingdom [6, 14, 23, 24]. However, this is the first study to examine the potential of using role substitution in a care home context as the primary model of care and it appears that many of the challenges of delivering care are similar.

## 5 Conclusion

The use or role substitution in a care home context offers promise, although sustained implementation raises a number of challenges both within the care home and for service planners. If the future challenges in this population group are to be met, an integrated approach across healthcare providers and regulators is essential to ensure that oral health is incentivised and given the priority that it requires across dependent settings.

## Author Contributions

A.H., G.T., G.M., S.R.B., I.C., C.S., C.O'N., R.E., A.K., K.M., F.S., M.D., C.L., L.M., K.S. and P.R.B. designed the trial and applied for funding from the N.I.H.R., P.R.B., S.R.B., G.T. and G.M. led the process evaluation and A.H. undertook all the interviews. P.R.B., S.R.B. and A.H. led the analysis. S.S.S., M.H. and A.J. completed the fieldwork for the main trial. A.H., P.R.B., S.R.B., G.T., G.M., S.S.S., M.H. and A.J. made a substantial contribution to the interpretation of data. A.H., P.R.B., S.R.B., G.M. and G.T. led the write‐up of the process evaluation and all authors subsequently reviewed the manuscript and provided final approval for it to be published. All authors agree to be accountable for all aspects of the work.

## Conflicts of Interest

The authors declare no conflicts of interest.

## Data Availability

The data that support the findings of this study are available on request from the corresponding author. The data are not publicly available due to privacy or ethical restrictions.
